# A continuum mechanics model for the Fåhræus-Lindqvist effect

**DOI:** 10.1007/s10867-021-09575-8

**Published:** 2021-07-03

**Authors:** Angiolo Farina, Fabio Rosso, Antonio Fasano

**Affiliations:** 1 Dipartimento di Matematica e Informatica “Ulisse Dini”, Università degli Studi di Firenze, Viale Morgagni 67/a, 50134 Florence, Italia; 2 FIAB S.p.A., Vicchio, Florence Italy; 3 I.A.S.I. – C.N.R., Via dei Taurini, 00185 Rome, Italy

**Keywords:** Blood flow in small vessels, Erythrocyte migration, Hemorheology, Mathematical modeling

## Abstract

The decrease in apparent relative viscosity that occurs when blood is made to flow through a tube whose diameter is less than about 0.3 mm is a well-known and documented phenomenon in physiology, known as the Fåhræus-Lindqvist effect. However, since the historical work of Fåhræus and Lindqvist (Amer. J. Physiol. **96**(3): pp. 562–568, 1931), the underlying physical mechanism has remained enigmatic. A widely accepted qualitative explanation was provided by Haynes (Amer. J. Physiol. **198**, pp. 1193–1200, 1960) according to which blood flows in microvessels with a core-annulus structure, where the erythrocytes concentrate within a central core surrounded by a plasma layer. Although sustained by observations, this conjecture lacks a rigorous deduction from the basic principles of continuum dynamics. Moreover, relations aimed to reproduce the blood apparent relative viscosity, extensively used in micro-circulation, are all empirical and not derived from the analysis of the fluid mechanical phenomena involved. In this paper, we apply the recent results illustrated in Guadagni and Farina (Int. J. Nonlinear Mech. **126**, p. 103587, 2020), with the purpose of showing that Haynes’ conjecture, slightly corrected to make it more realistic, can be proved and can be used to reach a sound explanation of the Fåhræus-Lindqvist effect based on continuum mechanics. We propose a theoretical model for the blood apparent relative viscosity which is validated by matching not only the original experimental data reported by Fåhræus and Lindqvist (Amer. J. Physiol. **96**(3), pp. 562–568, 1931), but also those provided by several subsequent authors.

## Introduction

The Fåhræus-Lindqvist (FL) effect is a phenomenon that occurs in blood vessels less than 0.3 mm in diameter and is named after the two Swedish scientists Robin Fåhræus and Johann Torsten Lindqvist [1]. It consists in a progressive reduction of the apparent relative viscosity of blood as the vessel radius decreases. Actually, such a phenomenon was almost simultaneously reported by Martini, Pierach, and Scheryer [2] and further investigated by Pries et al. [3], and by Secomb and Pries [4].

In experiments performed in glass tubes, Fåhræus and Lindqvist showed that the apparent relative viscosity of blood remains practically constant when the vessel diameter is larger than about 0.3 mm, but it keeps decreasing for lower and lower diameters. Therefore, 90 years ago, they concluded that [1, p. 568] “the viscosity of the blood is not a constant quantity, but dependent of the diameter of the tube.”

It has long been understood that this phenomenon originates from the non-uniform distribution of red blood cells (RBCs) over the cross-section of the tube. Indeed, a qualitative explanation, which received a large consensus, is the one proposed by Haynes [5] in the 1960s. According to Haynes’ interpretation, the distribution of RBCs on the vessel cross section changes when the blood flows in vessels with a sufficiently small diameter. More precisely, RBCs migrate towards the central part of the vessel (thus moving faster), while a less viscous layer of plasma (ideally free of RBCs) forms close to the walls. Hence, when blood flows in “small” vessels, the RBC volume fraction (hematocrit *ϕ*) varies over the cross section [6, 7]. For more details on Haynes’ theory and subsequent improvements, we refer the readers to Fournier [8], Chebbi [9, 10], Sharan and Popel [11], and Kumar and Graham [12]. Other numerous relevant references are listed in various papers (see, e.g., [13–16]) and books [17, 18].

Starting from the qualitative explanation of the FL effect by Haynes by means of a core-annulus flow structure, there was a considerable amount of research in this area. Indeed, it is known that deformable particles at a small Reynolds number migrate away from the vessel walls due to hydrodynamic interactions with the wall (see, e.g., [7, 19–22]). This phenomenon, also referred to as lift force, is responsible for the development of the core-annulus structure.

In this study, starting from a continuum mechanics approach, we show that the Haynes’ conjecture has, indeed, a rigorous physical base and it provides the starting point for a fluid dynamic interpretation of the FL effect. The key point is to treat blood, i.e., the plasma-RBC suspension, as an inhomogeneous viscous fluid whose apparent viscosity, denoted as^1^*η*^∗^, depends on the hematocrit *ϕ* (an aspect extensively treated in the literature) as well as on other quantities (e.g., shear rates). When *ϕ* is uniform (or almost uniform) over the flow domain, we recover the constitutive response of a homogeneous fluid, but when *ϕ* varies on the vessel cross section, such an inhomogeneity has a significant influence on the viscous stress, while it does not affect blood density *ρ*^∗^ (plasma and RBCs have practically the same density). For instance, Anand and Rajagopal [23] have shown how inhomogeneous fluids with viscosity that varies mildly but smoothly around a mean value may lead to dramatic differences in the rheological response.

According to the proposed approach, we treat the RBC plasma suspension as an inhomogeneous fluid. Of course, our theory is limited to those vessels whose diameter is sufficiently larger than the RBC dimensions. Indeed, when the vessel cross section becomes comparable with RBC maximum size, blood can hardly be considered a fluid amenable to the techniques of continuum mechanics [17, 24, 25]. Consequently, our model is not aimed at describing the so-called inverse FL effect, that is the increase of blood apparent viscosity in tubes whose cross section is comparable with RBC maximum size. Moreover, in capillary vessels, RBCs dispose themselves in a way such as to exploit the hydraulic pressure gradient, like a parachute [26]. In more detail, we model the hematocrit evolution by an advection equation in which any diffusive flux is ignored. Indeed, considering non-colloidal suspensions (the characteristic diameter of a RBC is sufficiently large for Brownian effects to be negligible) in the framework of mixtures theory, [27–29], the solid-fluid interaction typically depends on the difference between the phase velocities. However, writing the equations in a dimensionless form, the Darcy’s number Da (i.e., the square of the ratio between the macroscopic length scale and the particle dimension) appears in front of the solid-fluid interaction term. Since Da can become quite large, expanding the variables in terms of 1/Da, we find, at the leading order, that the two phases have the same velocity. Hence, if we limit ourselves to vessels whose cross section is sufficiently larger than the RBC diameter, we can assert with reasonable confidence that plasma and RBCs have practically the same velocity and the suspension can be considered a single non-homogeneous fluid (see, e.g., [30, 31]). Blood dynamics in such vessels is thus governed by the linear momentum balance equation, continuity equation, and the RBC conservation law, which simply reduces to the vanishing of the hematocrit material derivative.

Other approaches are possible for describing the evolution of *ϕ*. For instance, some authors (see, e.g., [10, 32]) considered the blood as a mono-modal suspension of plasma and RBCs, in which a diffusive processes may occur. According to such an approach, the net flux of particles consists of two contributions: a diffusive flux driven by the gradient of the shear rate and a diffusive flux due to the concentration gradient (with a diffusivity proportional to the local shear rate) [33, 34]. This model was used to predict the formation of a region close to the wall relatively poor in particles. By a suitable scaling approach, Leighton and Acrivos [33] and Pranay et al. [35] suggested that the flux of RBCs be driven by a concentration gradient with diffusivity proportional, in particular, to the shear rate. This approach, anyway, leads to a serious drawback when simple geometries and flow conditions are considered (such as steady Couette or Poiseuille motions). It turns out, indeed, that the hematocrit *ϕ* should be cusp-shaped at the centerline, a behavior never observed in real experiments. These difficulties can be overcome by means of a more sophisticated strategy as that recently developed by Monsorno et al. [36, 37], Lecampion and Garagash [38], Boyer et al. [39], and Ahnert et al. [40], where blood is treated as mixture. However, the related mathematical problem to solve shows a high degree of complexity mainly due to the boundary conditions to select.

In a recent paper by Ascolese et al. [13], Haynes’ conjecture was investigated on the basis of a mathematical model with the aim of clarifying the real influence of the FL effect on blood circulation. It was concluded that its advantage is not a reduction of power dissipation during the flow (as originally hypothesized by Haynes and several other authors since then), but, rather, a significant increase of blood discharge, thus largely improving tissues perfusion. In [13], it was also proved that power dissipation actually increases as the discharge increases.

In the present paper, we exploit the model by Ascolese et al. [13], but with a more basic purpose, namely to show that the FL effect has a precise fluid dynamical interpretation. Actually, our model, focussing on the flow in a tube, distinguishes two regions: an outer layer characterized by a “low” hematocrit and the complementary axisymmetric region to which most RBCs are segregated, where the hematocrit is constant and uniform. Such an approach differs slightly from the classical Haynes’ scheme in which the outer layer is cell free. Actually, there are many physical reasons for that and this modification allows improving the fit with experimental data. The two regions are separated by an interface, whose radius, denoted as *s*^∗^, is a function of the longitudinal coordinate *x*^∗^ along the vessel. From the point of view of continuum mechanics, *s*^∗^ is a material surface, i.e., a mathematical idealization. On the other hand, some modest diffusion, here neglected, will always be present, and this somehow makes *s*^∗^ not a net interface but rather a transitional layer.

Our analysis relies on the recent results by Guadagni and Farina [41], showing that the particle movement towards the duct axis can be explained as an entrance effect (i.e., in terms of the radial velocity arising in the entrance region of the vessel) and that the flow reaches very soon a core-annulus structure as the one considered here.

A key step is to define the outer layer thickness at the very entrance of the vessel, i.e., the difference between the vessel radius *R*^∗^ and $ s^{\ast }|_{x^{\ast }=0}=s_{0}^{\ast }$. Though numerous drift phenomena, linked to the collective RBCs dynamics taking place at the very entrance of the vessel, contribute to forming the marginal layer, in the literature, this is mainly attributed to the so-called “size exclusion effect” [7, 18, 42], i.e., most of the particles are excluded from a zone near the wall as a result of their finite “size.” Concerning RBCs, which have a discoid shape, such a size should not exceed the discoid maximal half thickness, in view of the fact that RBCs enter the vessel in a configuration in which they offer the least surface in the flow direction. Thus, denoting by *a*^∗^ the size of this layer, i.e., $a^{\ast }=R^{\ast }-s_{0}^{\ast }$, it is reasonable to guess that *a*^∗^ is going to depend on the geometrical properties of the RBCs in the considered sample, thus a quantity whose value cannot be given a priori with great accuracy. We then relate $ s_{0}^{\ast }$ to the asymptotic value to which *s*^∗^ stabilizes (denoted as $s_{\infty }^{\ast }$) and, applying the machinery of Ascolese et al. [13], we obtain the global blood apparent viscosity as a function of *R*^∗^, *a*^∗^ and of the central core viscosity which, in turn, depends on *ϕ*. The result fits the original experimental curves by Fåhræus and Lindqvist [1], thus providing an explanation of the FL effect just based on the basic principles of the dynamics of suspensions. We also consider other series of data examined in the relevant literature, i.e., the data by Kümin [43] and by Zilow and Linderkamp [44]. We however remark that our model, besides providing a theoretical justification of the FL effect, is also able to reproduce adequately the curves by Pries et al. [45] and by Secomb [4]. It is important to emphasize that these curves are based upon empirical formulas and have been obtained by fitting a large set of experimental data available in the literature, not upon the principle of fluid dynamics.

Of course, the model is obviously simplified since we have disregarded phenomena such as the RBC elastic properties [20, 46], the interaction between RBCs and the vessel endothelium (see, e.g., [47, 48]), and the viscoelastic properties of the plasma [49], which may play a role in the RBC collective dynamics.

The plan of the paper is as follows. In Section 2, we set down the basic equations and describe the mathematical model. In Section 3, we compare the downstream marginal layer thickness, i.e., $ R^{\ast }-s_{\infty }^{\ast }$, with the experimental data by Maeda et al. [50] and with those of Kim et al. [51]. In Section 4, we consider the classical empirical formulas by Pries et al. [45] and by Secomb [4] and compare our theoretical model with them. Sections 5 and 6 are devoted to the comparison with the experimental data by Fåhræus and Lindqvist and other authors. Concluding remarks are reported in the last section.

## The mathematical model

We consider a mechanically incompressible stationary laminar flow in a cylindrical vessel of radius *R*^∗^ and diameter *D*^∗^ = 2*R*^∗^. We denote by *x*^∗^, *r*^∗^ the longitudinal and radial coordinates, respectively, and by $\boldsymbol {v}^{\ast }=v^{\ast } \boldsymbol {e}_{x}+u^{\ast }\boldsymbol {e}_{r}$, the velocity field. In particular, the inlet discharge *Q*^∗^ is prescribed and we set $\left . \boldsymbol {v}^{\ast }\right \vert _{x^{\ast }=0}=V^{\ast }\boldsymbol {e}_{x}$, with *V*^∗^ = *Q*^∗^/*πR*^∗2^.

We model the blood as a solid-fluid mixture, whose components (RBCs and plasma) are incompressible in their pure states and have the same velocity and density. Thus, the mixture can be treated as a single inhomogeneous mechanically incompressible fluid whose dynamical state is specified by three dependent variables: hematocrit *ϕ* (volume fraction occupied by RBCs), velocity ***v***^∗^, and pressure *p*^∗^. Hence, in a general 3D context, the flow is governed by these equations:

1 $$ {\displaystyle{\frac{\partial \phi }{\partial t^{\ast }}}}+\nabla^{\ast }\cdot \left( \boldsymbol{v}^{\ast }\phi \right) =0,  $$

2 $$ \nabla^{\ast }\cdot \boldsymbol{v}^{\ast }=0,  $$

3 $$ \rho^{\ast }\left( {\displaystyle{\frac{\partial \boldsymbol{v}^{\ast }}{ \partial t^{\ast }}}}+\left( \boldsymbol{v}^{\ast }\cdot \nabla^{\ast }\right) \boldsymbol{v}^{\ast }\right) =-\nabla^{\ast }p^{\ast }+\nabla^{\ast }\cdot \mathbb{T}^{\ast },  $$

where *ρ*^∗^ is the blood density (constant and uniform), and $ \mathbb {T}^{\ast }$ is the non-spherical part of the Cauchy stress tensor whose constitutive equation is

4 $$ \mathbb{T}^{\ast }=\eta^{\ast }\left( \nabla^{\ast }\boldsymbol{v}^{\ast }+\nabla^{\ast }\boldsymbol{v}^{\ast \ T}\right) ,  $$

with *η*^∗^ viscosity of the mixture (mainly depending on both hematocrit and shear rate). Working in the context of mixture theory, (1) is the RBC conservation law which, when combined with (2), can also be written as

$$ {{\frac{\partial \phi }{\partial t^{\ast }}}}+\boldsymbol{v}^{\ast }\cdot \nabla^{\ast }\phi =0, $$

i.e., the material derivative of *ϕ* equal to 0. We are, indeed, assuming that plasma and RBCs have the same velocity, namely that the RBC diffusive velocity with respect to the mixture is negligible (see [27], Sect. 1.2). Equation (2) expresses the mass conservation for the mixture as a whole or, since plasma and RBCs are incompressible in their pure state, the volume additivity constraint (see [29], Sect. 2.8). Finally, (3) is the momentum balance equation for the mixture as a whole (treated as an inhomogeneous fluid), where body forces have been neglected. The model (1)–(3) is not complete because it contains an undetermined quantity, namely $\mathbb {T}^{\ast }$. The structure of $\mathbb {T}^{\ast }$ is given by the constitutive law (4), which satisfies both the frame-indifference requirement and the entropy inequality. Concerning blood viscosity, we set $\eta ^{\ast }=\eta _{p}^{\ast }\eta $, where $\eta _{p}^{\ast }$ is the plasma viscosity and *η* > 1, dimensionless, is usually referred to as the relative viscosity.

According to Haynes’ marginal theory [5] and following the same approach of [41] and [13], most RBCs are confined within a streamtube, also known as central core, whose radius *s*^∗^ (introduced earlier) evolves longitudinally, i.e., $s^{\ast }=s^{\ast }\left (x^{\ast }\right ) $. Next, still following [13] and [41], we consider the step-shaped profile for *ϕ*, i.e., *ϕ* = *ϕ*_*c*_ within the inner core and *ϕ* = *ϕ*_*A*_ in the outer layer. In particular, *ϕ*_*A*_ is significantly smaller than *ϕ*_*c*_ and both *ϕ*_*c*_, *ϕ*_*A*_ are uniform in space. However, in our approach, we do not make use of the hematocrit values, but we rather refer to the viscosities of the two domains.

**Fig. 1:**
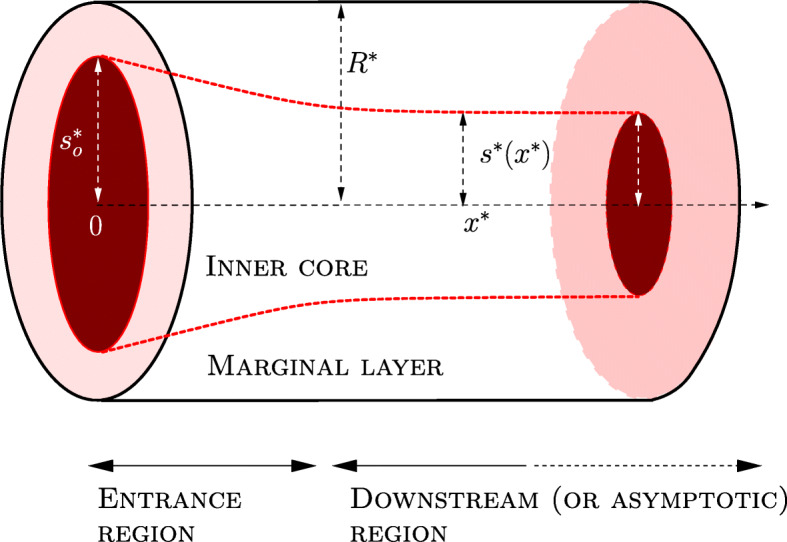
A schematic drawing of the geometrical model (the thickness of the outer layer *R*^∗^− *s*^∗^ is exaggerated for visualization purposes). In fluid dynamic terms, $r^{\ast }=s^{\ast }\left (x^{\ast }\right ) $ represents the streamtube whose radius, in *x*^∗^ = 0, is $ s_{0}^{\ast }=R^{\ast }-a^{\ast }$ (with *a*^∗^ average RBC half-thickness). This tubular region (surrounded by streamlines) forms the inner core where most of the RBCs are confined. In the entrance region, the flow has a radial component, pointing towards the axis, that drives the RBCs towards the central core. Downstream, i.e., in the asymptotic region, the radial velocity has vanished and the flow stabilizes into a core-annulus structure (see Guadagni and Farina [41] for more details). Although the size of the core decreases as we move away from the vessel inlet, its hematocrit remains constant because the erythrocytes increase their longitudinal velocity as they approach the axis

The central core shape is characterized by its radius $s_{0}^{\ast }$ at *x*^∗^ = 0 (an issue that we will discuss in the detail in the sequel) and by the peculiar evolution in space of *s*^∗^ caused by the curvature of the flow lines. Indeed, in [41], the authors show that in the entrance region the flow has a radial component (rapidly vanishing downstream) which, by shifting the particles towards the vessel axis, increases the thickness of the outer layer. Thus, the central core radius decreases from $s_{0}^{\ast }$ to an asymptotic limit, denoted by $s_{\infty }^{\ast }$, which, anyway, is reached relatively close to the inlet. Moreover, also the velocity field converges quickly to a pure axial flow, i.e., to $\boldsymbol {v}_{\infty }^{\ast }=v_{\infty }^{\ast }\boldsymbol {e}_{x}$. In this connection, we remark that some authors assume that *s*^∗^ is constant all over the whole length of the vessel and that the velocity field has only the longitudinal component [42]. Indeed, as shown by Guadagni and Farina in [41] in the framework of the Prandtl boundary layer theory [52], deriving the actual shape of the *s*^∗^ interface (schematically depicted in Fig. 1) and the flow radial and longitudinal components is, by far, not trivial. We also remark that in [41] a different geometrical setting was considered (a flow between two parallel plates). However, for our purposes, we are not going to use the actual shape of the interface, but just the fact that it stabilizes to the limit $s_{\infty }^{\ast }$, and this can be borrowed from the quoted paper.

We set

5 $$ \eta^{\ast }=\left\{ \begin{array}{lll} \eta_{p}^{\ast }\eta_{c}, & & 0\leq r^{\ast }\leq s^{\ast }, \\ & & \\ \eta_{p}^{\ast }\eta_{A}, & & s^{\ast }\leq r^{\ast }\leq R^{\ast }, \end{array} \right.  $$

where *η*_*c*_ > 1 is the core dimensionless relative viscosity and *η*_*A*_ ≥ 1 is the annulus relative viscosity. In particular, as it will be clarified in the sequel, we expect *η*_*A*_ to be definitely lower than *η*_*c*_ and slightly larger than 1. The idea, indeed, is to generalize Haynes’ approach [5], where *η*_*A*_ = 1.

The current literature reports numerous empirical formulas expressing the relative viscosity as a function of hematocrit (see, for example, [8, 53–59]). All these formulas are characterized by the fact that *η* is, in particular, an increasing function of *ϕ*. However, it must be pointed out, that, only at sufficiently large shear rates (i.e., larger than approximately 500s^− 1^), *η* can be safely approximated as a function depending only on *ϕ*. Indeed, in this limit, the relative viscosity tends to a value depending only on the hematocrit, and blood behaves as a Newtonian viscous fluid. For shear rates smaller than about 500s^− 1^, the blood viscosity starts depending on the shear rate too (see [17]). In small vessels, where, according to Secomb [7], the shear rate is usually less than 500 s^− 1^, the relative viscosity shows an evident dependence on both shear rate and hematocrit, thus leading to an extremely complex rheology. Therefore, in order to bypass this obstacle, we preferred to treat *η*_*c*_ as a parameter directly deducible from experimental data, rather than trying to guess any link to hematocrit and other quantities.

Concerning *η*_*A*_, we set

6 $$ \eta_{A}=1+\alpha \left( \eta_{c}-1\right) ,\ \ \ \text{with}\ \ \ \ 0\leq \alpha \ll1,  $$

and take *α* as a fitting parameter. We remark that *α* = 0 corresponds to an outer layer free from RBCs.

At the vessel entrance, the core radius, denoted as $s_{0}^{\ast }$ (see again Fig. 1), can be estimated in terms of the RBCs dimension, since, on average, RBCs cannot physically approach the vessel wall due to their finite size. So, we estimate $s_{0}^{\ast }=R^{\ast }-a^{\ast }$, with *a*^∗^ average RBC half-thickness. Concerning human RBCs, their minimum thickness in a disk-like configuration is 2 − 3 μm [60]. We use *a*^∗^ as a fitting parameter but we limit its values in a physically acceptable range, i.e., 1 μm ≤ *a*^∗^≤ 1.5 μm, roughly corresponding to RBC half-thickness. We remark that the marginal layer is not completely devoid of RBCs.^2^ A reasonable explanation is due to the fact that the marginal exclusion effect cannot be precisely stated (as it would be if the RBCs were rigid spheres) and it should be more understood as a statistical concept (see, for example, [42], pp. 145–146). Thus, a certain amount of RBCs is present in the marginal layer and this value might have some variability. However, the outer layer hematocrit is expected to be remarkably smaller than that in the core and it is accounted for by the parameter *α*, which, in turn, is expected to be smaller than 1 (typically, not exceeding 0.15). In particular, to simplify this complex dynamics, we consider the outer layer viscosity uniform and expressed by (6).

Downstream, as proved in [41], both the core radius and the velocity field stabilize to asymptotic values, i.e., $s_{\infty }^{\ast }$ and $\boldsymbol {v}_{\infty }^{\ast }=v_{\infty }^{\ast }\left (r^{\ast }\right ) \boldsymbol {e}_{x}$, since the RBC radial displacement caused by the radial velocity quickly vanishes outside the entrance region. We indeed assume that the total vessel length is much larger than the entrance length (i.e., the length until the flow is converged) so that the flow has reached its asymptotic profile well before the blood flows out. Recent papers [61, 62] investigate the effect of the entrance region on flow resistance and on RBC marginalization. However, these studies refer to vessels whose diameter is comparable with the maximum size of RBC, i.e., to vessels where the continuum approach that we propose cannot be safely applied.

The key point of the model is that there is a relationship between $ s_{\infty }^{\ast }$ and $s_{0}^{\ast }$. Indeed, exploiting the continuity equation, we can write

7 $$ \pi V^{\ast }s_{0}^{\ast^{2}}=2\pi {\int}_{0}^{s_{\infty }^{\ast }}v_{\infty }^{\ast }\left( r^{\ast }\right) r^{\ast }dr^{\ast },  $$

where $v_{\infty }^{\ast }\left (r^{\ast }\right ) $ is the classical core-annulus flow. Hence, introducing the dimensionless variables *r* = *r*^∗^/*R*^∗^, $s_{\infty }=s_{\infty }^{\ast }/R^{\ast }$, $ s_{0}=s_{0}^{\ast }/R^{\ast }=1-a^{\ast }/R^{\ast }$, *v* = *v*^∗^/*V*^∗^, and slightly extending the procedure presented in Ascolese et al. [13], we have

8 $$ v_{\infty }\left( r\right) =\frac{2}{\frac{s_{\infty }^{4}}{\eta_{c}}+ \frac{1}{\eta_{A}}\left( 1-s_{\infty }^{4}\right) }\times\left\{ \begin{array}{lll} \frac{s_{\infty }^{2}-r^{2}}{\eta_{c}}+\frac{1-s_{\infty }^{2}}{\eta_{A}} , & & \text{if} 0\leq r\leq s_{\infty }, \\ & & \\ \frac{1-r^{2}}{\eta_{A}}, & & \text{if} s_{\infty }\leq r\leq 1. \end{array} \right.  $$

Then, exploiting (8), (7) can be rewritten as

9 $$ {s_{0}^{2}}={\displaystyle{\frac{1}{s_{\infty }^{4}\left( {\displaystyle{\frac{ 1}{\eta_{c}}}}-{\displaystyle{\frac{1}{\eta_{A}}}}\right) +\frac{1}{\eta_{A}}}}\left[ s_{\infty }^{4}\left( {\displaystyle{\frac{1}{\eta_{c}}}}- \frac{2}{\eta_{A}}\right) +\frac{2s_{\infty }^{2}}{\eta_{A}}\right] ,}  $$

which is a fourth-order algebraic equation in $s_{\infty }$, having only one physically significant solution, i.e., $s_{\infty }\in \left (0,1\right ) $,

10 $$ s_{\infty }=\frac{s_{0}}{\sqrt{1+\sqrt{\left( 1-{s_{0}^{2}}\right) \left[ 1-{s_{0}^{2}}\left( 1-\frac{\eta_{A}}{\eta_{c}}\right) \right] }}}.  $$

**Fig. 2:**
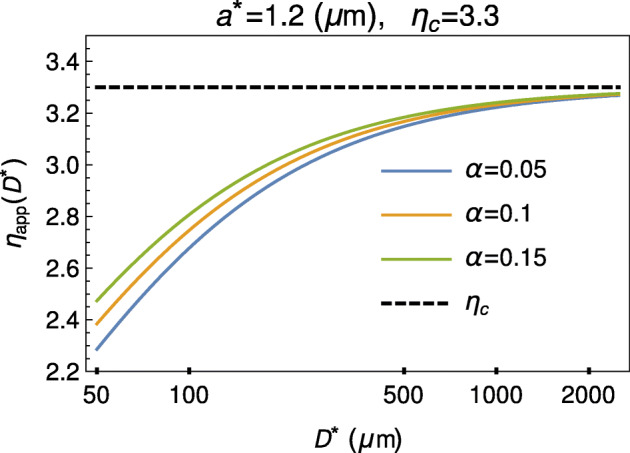
Relative apparent viscosity (continuous curves) of blood given by (11) as a function of tube diameter *D*^∗^, for *η*_*c*_ = 3.3 (dashed line), *a*^∗^ = 1.2*μ* m and *α* = 0.05,0.1,0.15

To compute the dimensionless asymptotic apparent blood viscosity, we exploit the classical Poiseuille formula for the discharge referred to the flow as a whole [13], getting

11 $$ \eta_{\text{app}}=\frac{\eta_{\text{app}}^{\ast }}{\eta_{p}^{\ast }}= \frac{1}{s_{\infty }^{4}\left( \frac{1}{\eta_{c}}-\frac{1}{\eta_{A}} \right) +\frac{1}{\eta_{A}}}.  $$

with *η*_*A*_ given by (6). We have thus obtained a formula for the apparent viscosity as a function of the core and outer annulus viscosities *η*_*c*_, *η*_*A*_, and, through *s*_0_ = 1 − 2*a*^∗^/*D*^∗^, of the vessel diameter *D*^∗^ = 2*R*^∗^ and the gap *a*^∗^. Figure 2 shows the typical behavior of *η*_app_ given by (11) as a function of *D*^∗^

In particular, Fig. 2 clearly highlights that (11) reproduces these two fundamental properties (see, for example [26]): the reduction of apparent viscosity of blood in “narrow” vessels and its asymptotic behavior for “large” *D*^∗^. Indeed, as the tube diameter decreases below about 500 μm, the apparent viscosity declines to levels substantially lower than the so-called bulk viscosity. Moreover, Fig. 2 provides also the physical meaning of *η*_*c*_: it is the asymptotic limit of *η*_app_, i.e., the bulk viscosity. Indeed, in vessels where 2*a*^∗^/*D*^∗^≪ 1, we have $s_{0}\rightarrow 1$, and consequently also $ s_{\infty }\rightarrow 1$, meaning that the core practically occupies the whole cross section. The Newtonian case is thus recovered because the outer layer is so thin with respect to the vessel size to have any significant effect on the overall blood dynamics.

## Asymptotic outer layer thickness

**Fig. 3:**
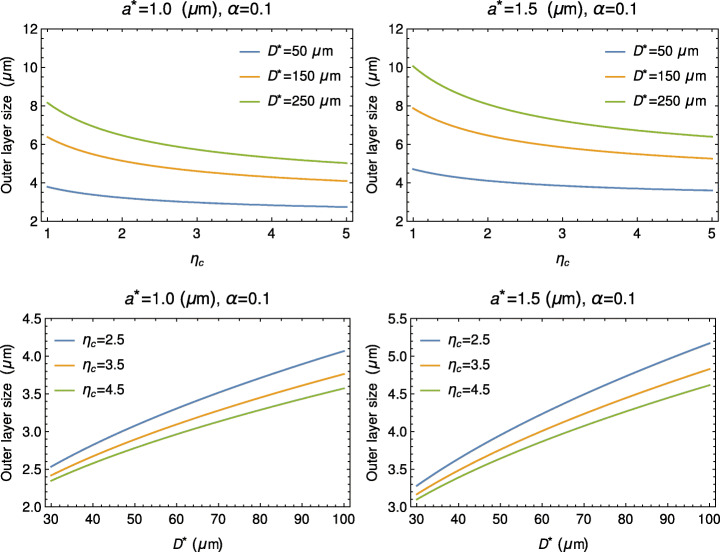
Upper panels: behavior of $R^{\ast }-s_{\infty }^{\ast }({\eta }_{c}) $ for given *D*^∗^. Lower panels: behavior of $R^{\ast }-s_{\infty }^{\ast }\left (D^{*}\right ) $ for given *η*_*c*_. In all cases, for given *α* and *a*^∗^, the thickness of the marginal layer is a decreasing function of *η*_*c*_ and an increasing function of *D*^∗^. By increasing *a*^∗^, the corresponding size of the marginal layer is significantly higher. The effect of changing *α* even one order of magnitude is rather modest and it is not shown

Equation (10) allows estimation of $s_{\infty }$, and so the asymptotic marginal layer thickness, i.e., $R^{\ast }-s_{\infty }^{\ast }$, which we can compare with the experimental values. Figure 3 shows the behavior of $\ R^{\ast }-s_{\infty }^{\ast }$, either as a function of *η*_*c*_ for given *D*^∗^, or as a function of *D*^∗^ for given *η*_*c*_. By comparing panels on the left side with the corresponding ones on the right side, we see that the effect of increasing *a*^∗^ is a significant increase of the size thickness.

Actually, it is difficult to find direct experimental measurements of the outer layer thickness for cylindrical vessels whose diameter is about 100 μm or larger. Experimental data referring to smaller diameters are available in Fig. 5 of [50]. There, the authors consider hematocrit values in the range 0.08–0.45, and a tube diameter *D*^∗^ = 30 ± 5 μm. Although the conditions of the experiment are outside the range of our theory, we compare the outer layer size $R^{\ast }-s_{\infty }^{\ast }$ with the experimental values reported in Fig. 5 of [50]. These data provide only the discharge hematocrit but neither the apparent viscosity *η*_app_ nor *η*_*c*_. Since the core relative viscosity is a necessary input of (10), we need an estimate (which may even be coarse) of *η*_*c*_ in terms of the only available data, i.e., *ϕ*. We therefore select Charm and Kurland’s empirical formula [54]

12 $$ \eta_{{~}_{\text{CK}}}(\phi )=\left[ 1-\phi \left( 0.07\exp \left( 2.49 \phi +\frac{1107}{310}\exp (-1.69 \phi )\right) \right) \right]^{-1}.  $$

and use it just to estimate *η*_*c*_, in terms of *ϕ*. Indeed, it should be considered that the shear stress in the core is reduced due to the presence of the boundary layer. Of course, other empirical formulas linking *η*_*c*_ and *ϕ* can be selected [55, 56, 58, 63, 64]. However, they give similar values of $\left (R^{\ast }-s_{\infty }^{\ast }\right ) $. The other two parameters appearing in (10), i.e., *α* and *a*^∗^, have been taken as 0.1 and 1.2 μm, respectively. We selected *a*^∗^ = 1.2 μm, since the data reported in [50] refer to human blood. Table 1 shows the size of the marginal layer measured by Maeda et al. [50] when *ϕ* = 0.08,0.16,0.3,0.45 and the size obtained using (10). Although Maeda et al. report the data referring to two different types of tube, namely elastic and rigid, in Table 1, we consider only the “rigid” case. Small changes of *α* and *a*^∗^ do not modify significantly the outer layer size which remains comparable with the measures by Maeda et al. [50].

**Table 1:** Thickness of the marginal layer obtained using (10) with *a*^∗^ = 1.2μm, *α* = 0.1 (fourth column), and the values measured by Maeda et al. [50] (third column) when *ϕ* = 0.08,0.16,0.3,0.45 in tubes of diameter 30μm. The value of *η*_*c*_ appearing in (10) has been estimated using (12)

*ϕ*	*η*_CK_ [−]	$(R^{*}-s_{\infty }^{\ast })$ (meas.)	$(R^{*}-s_{\infty }^{\ast })$ (theor.)
0.08	1.18	3.9 ± 1.0 μm	2.7 μm
0.16	1.34	3.1 ± 0.5 μm	2.7 μm
0.30	1.61	2.3 ± 0.8 μm	2.6 μm
0.45	2.05	1.6 ± 0.5 μm	2.6 μm

Other sets of reliable data are due to Kim et al. [51]. In Table 2, we compare the thickness of the cell-poor layer reported in [51] with the theoretical values predicted by the mathematical model.

**Table 2:** Thickness of the marginal layer obtained using (10) with *a*^∗^ = 0.8μm, *α* = 0.1, and the values measured by Kim et al. [51]. The value of *η*_*c*_ appearing in (10) has been estimated again using (12). The experiments reported in [51] refer to rat blood, whose RBCs are 25–30% smaller than the human ones (see [65]), which justifies *a*^∗^ = 0.8 μm

	*D*^∗^	$(R^{*}-s_{\infty }^{\ast })$	$(R^{*}-s_{\infty }^{\ast })$
		(meas.)	(theor.)
*ϕ* = 0.42, $\eta _{{~}_{\text {CK}}}=1.94$	72.3 μm	2.7 ± 0.5 μm	3.4 μm
	49.2 μm	3.1 ± 0.6 μm	2.9 μm
	45.3 μm	2.3 ± 0.4 μm	2.8 μm
	30.8 μm	2.1 ± 0.4 μm	2.4 μm
*ϕ* = 0.41, *η*_CK_ = 1.90	71.1 μm	3.2 ± 0.7 μm	3.2 μm
	60.2 μm	2.2 ± 0.4 μm	3.0 μm
	54.2 μm	2.9 ± 0.6 μm	2.9 μm
	51.7 μm	2.3 ± 0.4 μm	2.8 μm

An updated reference concerning measurements of the outer layer can be found in the PhD thesis by Gliah [66]. However, experimental conditions, type of blood used, size and shape of the vessels, and measurement techniques vary so much in the current literature that an exhaustive comparison is very difficult.

For the purpose of the present research, it suffices to say that, in all cases, we obtained values of the outer layer thickness very similar to the experimental ones reported in Tables 1 and 2.

Recalling also the great uncertainty of the available data and that we are at the edge of applicability of the continuum hypothesis, the theoretical values of the outer layer thickness shown in Tables 1 and 2 are encouraging.

## Comparison with classical empirical formulas

Pries et al. [45] provided the following empirical formula to evaluate *η*_app_

13 $$ \eta_{\text{app}}^{(P)}(d,\phi )=1+\left( \eta_{0.45}^{(P)}(d)-1\right) \frac{(1-\phi )^{ c(d)}-1}{(1-0.45)^{ c(d)}-1},  $$

where

14 $$  \eta_{ 0.45}^{(P)}(d)= 220\exp(-1.3 d)+3.2-2.44\exp\left( -0.06 d^{ 0.645} \right), $$

and

15 $$  c(d)= (0.8+\exp(-0.075 d))\left( \frac{1}{1+10^{-11}d^{ 12}} -1 \right)+ \frac{1}{1+10^{-11}d^{ 12}}. $$

A similar empirical formula was suggested by Secomb [4]

16 $$  \eta_{\text{app}}^{(S)}(d,\phi)=\frac{d^{ 2} \left( \frac{d^{ 2} (\eta_{0.45}^{(S)}(d)-1) \left( (1-\phi)^{ c(d)}-1\right)}{(d-1.1)^{2} \left( 0.55^{ c(d)}-1\right)}+1\right)}{(d-1.1)^{2}}, $$

where

17 $$ \eta_{ 0.45}^{(S)}(d)=3.2 -2.44\exp \left( {-0.06 d^{ 0.645}}\right) +6\exp \left( {-0.085 d}\right) .  $$

In all these relations, *d* denotes the tube diameter (*D*^∗^ in our notation) which is measured in micrometers. These formulas are both based on a best fitting procedure using several sets of data collected in the last 70 years.

**Fig. 4:**
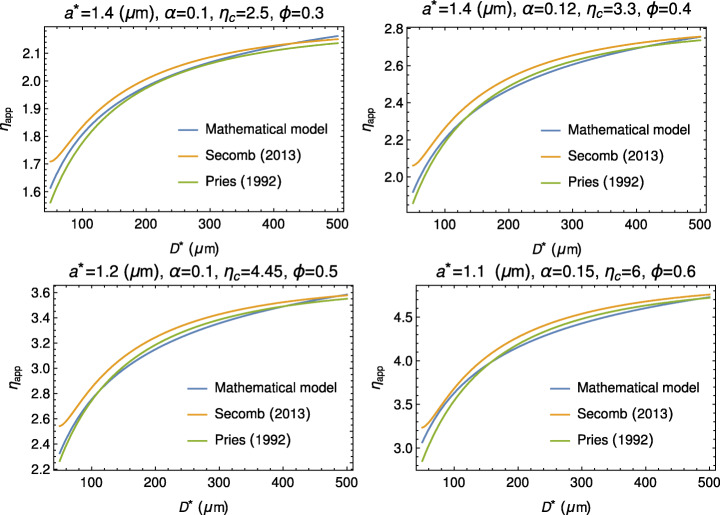
Comparison between the mathematical model (11), (10) and the empirical formulas by Pries and Secomb for four different values of the hematocrit. On the top of each plot, the values of *a*^∗^, *α*, *η*_*c*_, and the hematocrit *ϕ* used in formulas (13)–(17)

It is interesting, anyway, to compare both these empirical formulas with our model. As Fig. 4 shows, the agreement is satisfactory.

The values of *a*^∗^ range between 1.1 and 1.4 μm, which are compatible with RBC half-thickness in a disk-like configuration. Moreover, we point out that the marginal layer does not consist of pure plasma. Referring to the cases *ϕ* = 0.4–0.5, we have, on average, *α* ≈ 0.11, which, according to (6), entails *η*_*A*_ ≈ 1.2. Now, if we estimate the corresponding hematocrit using, for example, (12) we get that *ϕ* in the outer layer ranges around 0.13, remarkably smaller, as expected, than the one of the inner core.

## Comparison with the data of Fåhræus and Lindqvist

**Fig. 5:**
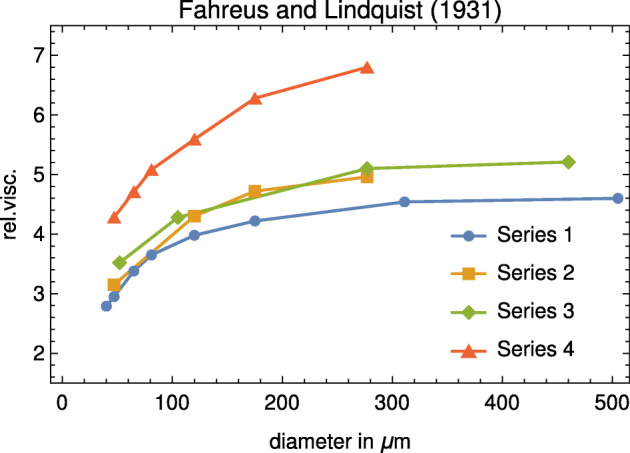
The original data by Fåhræus and Lindqvist published in 1931: series 1, 2, and 4 refer to Lindquist’s blood, while series 3 refers to Fåhræus’ blood

We decided to devote a single section to the celebrated article by Fåhræus and Lindqvist [1]. Figure 5 shows graphically the original data reported in [1].

**Fig. 6:**
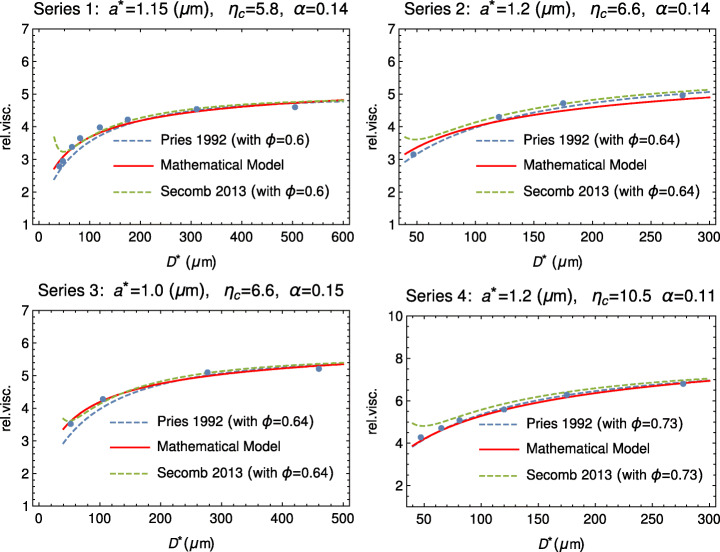
Comparison between the experimental series 1, 2, 3, and 4 reported in Tables at p. 565 of Fåhræus and Lindqvist [1] (dots) and the theoretical model (11) (solid curve). The fitting via the empirical formulas (13) by Pries and (16) by Secomb is also shown (dashed curves). On the top of each plot, the values of *a*^∗^, *α*, *η*_*c*_ and the hematocrit *ϕ* used in formulas (13)–(17) to fit the Fåhræus and Lindqvist data

In Fig. 6, we reported the four experimental series of data for the apparent relative viscosity documented in the original paper by Fåhr æus and Lindqvist [1] along with the function $\eta _{\text {app} }\left (D^{\ast }\right ) $ given by (11).

We stress that our estimates of *a*^∗^ are still falling in an acceptable range and they look rather stable. We also emphasize that the experimental data do not refer to the same human blood (series 1, 2, and 4 refer to the Lindquist’s blood, while series 3 refers to Fåhræus’ blood) and that they are certainly affected by an experimental error which, unfortunately, is not documented in the original paper [1]. In particular, the experimental errors can range between 10 and 20% as shown, for example, in Table 1, on p. 596 of Zilow and Linderkamp [44].

We remark that to fit the FL data with the empirical formulas by Pries and Secomb, hematocrit values larger than 0.6 are required, resulting in high relative viscosities. In particular, series 4 corresponds to a very high level of *ϕ*, close to the so-called jamming transition (see, e.g., [17, 67]).

The fits just found provide also a theoretical explanation of the fact that the FL effect is observable for $D^{\ast }\lesssim 0.3~\upmu $m. Indeed, it is only below that threshold that the model indicates a non-negligible variation of viscosity.

## Comparison with other experimental data

In this section, we compare the theoretical model (11) with the data by Zilow and Linderkamp [44] and Kü min [43].

**Fig. 7:**
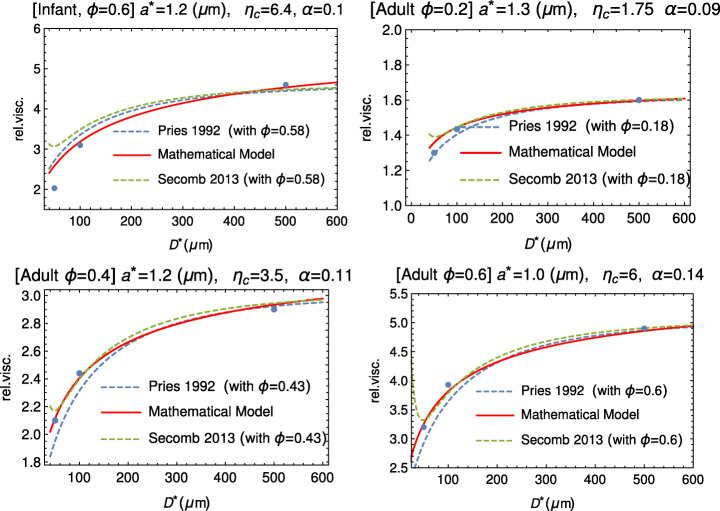
Comparison between our mathematical model (solid curve) and data by Zilow and Linderkamp [44] (dots). These authors considered both adult and infant blood samples at different values of the hematocrit. The empirical fitting via the empirical formulas (13) by Pries and (16) by Secomb is also shown (dashed curves)

Figure 7 shows the comparison between our model and the experimental data extracted from Fig. 1, at p. 597, of Zilow and Linderkamp [44] and referring to both adults and infant blood samples. In Table 3, we report *η*_*c*_ and *a*^∗^ for three values of the hematocrit.

**Table 3:** Values of *η*_*c*_, *α*, and *a*^∗^ used to fit the data extracted from Fig. 1, at p. 597, of Zilow and Linderkamp [44], referring to three different values of the hematocrit

	*ϕ*	*η*_*c*_ [–]	*a*^∗^(μm)	*α*
Infant	0.6	6.4	1.2	0.1
Adult	0.2	1.7	1.3	0.09
Adult	0.4	3.5	1.2	0.11
Adult	0.6	6.0	1.0	0.14

**Fig. 8:**
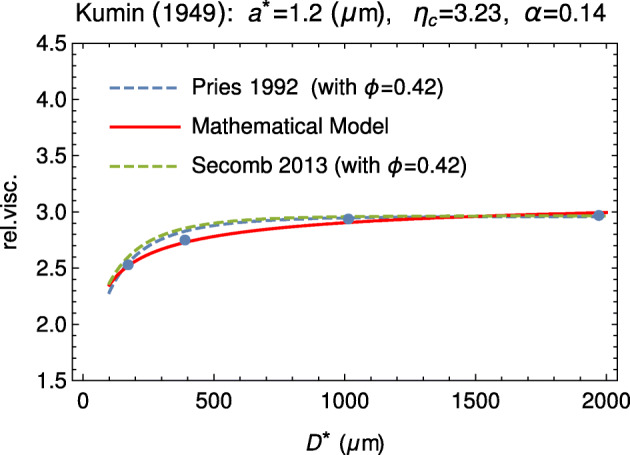
Comparison between our model and the experimental data by Kümin [43] (dots). Data are extracted from Fig. 2, at p. 1195 of [5]. The empirical fitting via the empirical formulas (13) by Pries and (16) by Secomb is also shown (dashed curves)

Figure 8 shows the comparison between our model and the experimental data by Kümin [43]. Data are extracted from Fig. 2, on p. 1195 of [5].

As in previous sections, in all figures, for completion, also the empirical curves of Pries and Secomb are shown (dashed lines). In all cases, the parameter *a*^∗^ is in agreement with the other cases considered here.

## Conclusions

The FL effect was discovered 90 years ago and ever since physicians have speculated about its meaning, its origin, and its purpose. The RBC segregation hypothesis, formulated 30 years later by Haynes, opened the way to an interpretation based on physical arguments. Haynes’ assumption was rather extreme: in small (though not too small) vessels, two flow regions are present, a marginal one free of RBCs and an internal one, in which all RBCs are concentrated. Based on this picture, Ascolese et al. [13] formulated a mathematical model and computed the corresponding two-phase steady unidirectional flow. In that way, they disproved the early conjecture that the FL effect could reduce power dissipation due to internal friction (which, on the contrary, increases) and concluded that the real advantage of the FL effect is an increase of blood discharge, which improves tissues perfusion, thus exploiting at the best the hydraulic gradient made available by the heart.

In the present paper, a finer analysis was performed aimed at retrieving, on a pure continuum mechanics basis, the experimental curves originally obtained by FL in their celebrated paper, relating the apparent blood viscosity to the vessel diameter. This goal has been achieved by correlating the thickness of the marginal layer at the vessel entrance with its asymptotic value, by using both the peculiar core-annular structure of the flow and a physically reasonable estimate of the inlet radius of the core and of the viscosities of both inner core and marginal layer. In fact, in the model, there are two fitting parameters, *a*^∗^ and *α*, for which both the physical meaning and the range in which they can vary are well specified. The first one is the minimum marginal layer thickness whose order of magnitude ranges around the RBCs half thickness (i.e., 1–1.5μm for human blood). The second one accounts for the outer layer viscosity (and consequently its relative hematocrit) with respect to the one of the central core. In particular, this means that we consider the marginal layer not completely free of RBCs. However, by specifying that $\alpha =\mathcal {O}\left (10^{-1}\right ) $, we require that the marginal layer viscosity is significantly lower than that of the central core and this obviously implies that the marginal hematocrit is significantly lower than the one of the core.

The physical background of the model is provided by the recent paper by Guadagni and Farina [41] in which the migration phenomenon in the flow of suspensions has been carefully investigated.

The present model provides a physical explanation of the FL effect, showing that this can be mainly attributable to the peculiar flow that develops in the vessel entrance region. Moreover, the model prediction fits the experimental results by Maeda et al. [50], Kim et al. [51], and the experimental curves by Fåhræus and Lindqvist [1], Kümin [43], and Zilow and Linderkamp [44] as well as the empirical formulas by Secomb [4] and Pries et al. [45].

Finally, we emphasize that the proposed model essentially relies on the estimate of the initial marginal layer thickness (indeed used as a fitting parameter) and on the assumption that the inner core and marginal layer viscosities are uniform (which implicitly entails that the hematocrit in the two regions is uniform). A conclusive theoretical advance would be an analysis of the entrance dynamics which does not use the Prandtl approximation and does not assume a uniform step-shaped hematocrit profile (as we did in the present work). Of course, this would provide a more accurate estimate of the RBC radial distribution at the inlet of the vessel and, consequently, of the asymptotic flow structure. However, the price to pay for this choice would be an increase of the model complexity that would imply giving up an explicit (and simple) formula for *η*_app_ as the one proposed here.

## Footnotes

1 Starred quantities are dimensional.

2 For instance, Kim et al. [51] refer to the the marginal layer as a cell-poor layer.
